# Transformational Leadership and Nurse Job Performance Through Self‐Efficacy in Chinese Tertiary Hospitals

**DOI:** 10.1002/nop2.70579

**Published:** 2026-04-29

**Authors:** Yuan Jiang

**Affiliations:** ^1^ Tenth People's Hospital of Tongji University Shanghai China

**Keywords:** China, JD‐R model, job performance, nursing leadership, self‐efficacy, structural equation modelling, transformational leadership

## Abstract

**Aim:**

To examine the associations among transformational leadership, nurse self‐efficacy and job performance in tertiary hospitals in Shanghai, China.

**Design:**

A cross‐sectional quantitative study guided by social cognitive theory and the Job Demands–Resources (JD‐R) model.

**Methods:**

Using convenience sampling, registered nurses were recruited from three tertiary Grade‐A public hospitals in Shanghai between September and November 2024. Eligibility criteria included holding a valid nurse licence, having at least 1 year of independent clinical experience, and being currently employed full‐time. Of 675 invitations distributed, 460 valid responses were included in the final analysis. Validated instruments were used to measure transformational leadership, nurse self‐efficacy and job performance. Data were analysed using SPSS 25.0 and AMOS 25.0. Confirmatory factor analysis and covariance‐based structural equation modelling were conducted using maximum likelihood estimation, and indirect effects were tested via bootstrapping with 5000 resamples.

**Results:**

Transformational leadership was positively associated with nurse self‐efficacy (*β* = 0.33, *p* < 0.001) and job performance (*β* = 0.18, *p* < 0.001). Nurse self‐efficacy was positively associated with job performance (*β* = 0.47, *p* < 0.001) and partially mediated the association between transformational leadership and job performance. The indirect effect was significant (*β* = 0.15, 95% CI [0.09, 0.21]) and accounted for 46.3% of the total effect. The final model demonstrated acceptable fit (CFI = 0.97, RMSEA = 0.05).

**Conclusion:**

Among nurses in three Shanghai tertiary public hospitals, transformational leadership was associated with higher self‐efficacy and better self‐reported job performance and self‐efficacy showed a statistically significant mediating role. Because the data were cross‐sectional and based on single‐source self‐report, the findings should be interpreted as associational rather than causal.

**Patient or Public Contribution:**

No patient or public contribution.

## 1 Introduction

Nurse job performance is closely related to care quality, teamwork, patient safety and organisational effectiveness in contemporary healthcare systems (Aung Po et al. 2024; Krijgsheld et al. 2022). As the largest group of frontline healthcare professionals, nurses undertake not only direct clinical care but also coordination, communication, patient education and advocacy. In high‐demand hospital environments, sustaining strong nursing performance is therefore a major management priority.

In China, tertiary Grade‐A public hospitals occupy a central position in urban healthcare delivery and typically manage high patient volumes, complex cases and intensive organisational demands (Chen et al. 2024; Fu et al. 2025). These hospitals are also characterised by relatively hierarchical administrative structures and strong performance expectations. Such conditions make them an important context in which to examine factors associated with nurse job performance, especially leadership and psychological resources.

Transformational leadership has received extensive attention in nursing and healthcare research because it emphasises vision, role modelling, individualised support and intellectual stimulation (Bass and Riggio 2006; Brewer et al. 2016; Niinihuhta et al. 2022; Ystaas et al. 2023). Studies from different countries have linked transformational leadership with favourable nurse‐related outcomes such as work engagement, job satisfaction, psychological safety, retention and performance (Boamah et al. 2018; Khan et al. 2022; Wang et al. 2021). However, the strength and interpretation of these associations may vary across institutional and cultural settings. In Chinese hospitals, where hierarchy, coordination and role boundaries are often prominent, leadership signals may carry particular salience for staff perceptions and work behaviour (Hu et al. 2025; Xie et al. 2020; Ying et al. 2021).

A potentially important explanatory factor is nurse self‐efficacy, defined as nurses' confidence in their capability to carry out work‐related tasks effectively. According to Bandura's social cognitive theory, self‐efficacy is a central determinant of motivation, persistence and behavioural regulation (Bandura 1991, 2001). Similarly, the JD‐R model conceptualises self‐efficacy as a personal resource that may support functioning under demanding work conditions (Bakker and Demerouti 2017; Demerouti et al. 2001). Prior nursing studies have shown that self‐efficacy is associated with performance, decision‐making and adaptation to clinical demands (Kallerhult Hermansson et al. 2024; Kim et al. 2022; Lee and Ko 2010).

Although these relationships are theoretically plausible, fewer studies have examined them together in Chinese tertiary hospital settings using an explicit mediation framework. Much of the available literature has been developed in non‐Chinese settings or has focused on direct effects only. This leaves room for further evidence on whether transformational leadership, nurse self‐efficacy, and job performance are linked in ways that are consistent with social cognitive theory and the JD‐R model in a hierarchical, high‐intensity hospital environment.

Accordingly, this study examined the associations among transformational leadership, nurse self‐efficacy and nurse job performance among nurses in three tertiary Grade‐A public hospitals in Shanghai, China. Specifically, the study tested whether nurse self‐efficacy showed a statistically significant mediating role in the relationship between transformational leadership and nurse job performance. By situating the analysis in a specific Chinese hospital context and adopting cautious associational inference, this study aims to contribute contextually grounded evidence for nursing leadership research and management practice.

## 2 Theoretical Framework

### 2.1 Theoretical Foundations

This study was guided by Bandura's social cognitive theory and the Job Demands–Resources (JD‐R) model. Social cognitive theory emphasises the role of self‐efficacy in shaping how individuals think, regulate effort and persist in the face of challenges (Bandura 1991, 2001). In nursing contexts, stronger self‐efficacy has been associated with greater confidence in clinical judgement, communication and task management (Kallerhult Hermansson et al. 2024; Lee and Ko 2010).

The JD‐R model proposes that employee functioning is shaped by the balance between job demands and job resources, and that personal resources can also support performance and well‐being (Bakker and Demerouti 2017; Demerouti et al. 2001). Leadership can be conceptualised as an important job resource because supportive leaders may clarify expectations, provide feedback and strengthen employees' psychological resources (Tummers and Bakker 2021).

Integrating these frameworks, transformational leadership was conceptualised in this study as an organisational resource associated with nurse self‐efficacy and job performance, while self‐efficacy was conceptualised as a personal resource associated with performance. This integrated perspective is especially relevant in tertiary hospital environments where work demands are high and leadership structures are salient.

### 2.2 Transformational Leadership, Self‐Efficacy and Job Performance

Transformational leadership includes idealised influence, inspirational motivation, intellectual stimulation and individualised consideration (Bass and Riggio 2006). In nursing settings, such leadership behaviours have been associated with better staff outcomes and more positive work environments (Boamah et al. 2018; Brewer et al. 2016; Ystaas et al. 2023). In Chinese tertiary hospitals, these associations may be particularly meaningful because leadership behaviours may also be interpreted as signals of institutional support and legitimacy (Hu et al. 2025; Xie et al. 2020).

Nurse self‐efficacy reflects confidence in managing patient care, communication, problem‐solving and professional responsibilities. Prior evidence suggests that nurses with stronger self‐efficacy tend to report more adaptive functioning and better work‐related outcomes (Kim et al. 2022; Lee and Ko 2010). Nurse job performance, in turn, includes work coordination, work engagement and effective completion of clinical responsibilities (Sarıköse and Göktepe 2022; Wang et al. 2015).

Based on the above frameworks and literature, the following hypotheses were tested:

Hypothesis 1 *Transformational leadership is positively associated with nurse self‐efficacy*.

Hypothesis 2 *Nurse self‐efficacy is positively associated with job performance*.

Hypothesis 3 *Transformational leadership is positively associated with job performance*.

Hypothesis 4 *Nurse self‐efficacy partially mediates the association between transformational leadership and job performance*.

### 2.3 Conceptual Model

**FIGURE 1:**
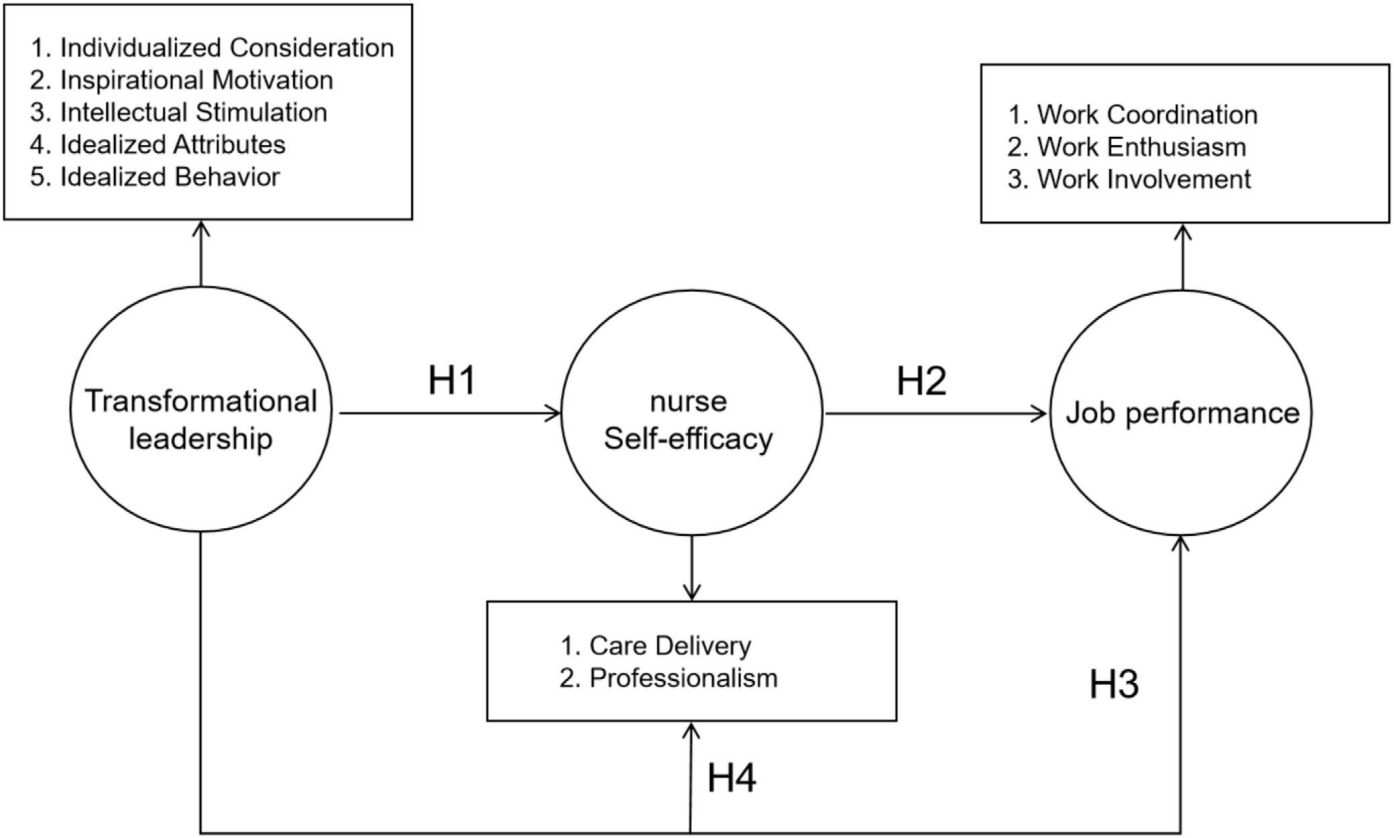
Conceptual model of hypothesised relationships.

The conceptual model is shown in Figure 1. The conceptual model was developed by the authors based on Bandura's social cognitive theory, the JD‐R model, and prior empirical research on transformational leadership, self‐efficacy and work outcomes (Bandura 1991; Demerouti et al. 2001; Katou et al. 2022; Tummers and Bakker 2021).

## 3 Methods

### 3.1 Research Design

This study used a quantitative cross‐sectional design to examine the associations among transformational leadership, nurse self‐efficacy and job performance. The design was suitable for testing the hypothesised measurement and structural relationships using confirmatory factor analysis (CFA) and structural equation modelling (SEM). However, because all variables were measured at one time point using self‐report data, the study supports associational rather than causal inference.

### 3.2 Setting, Hospital Selection and Sampling

A convenience sampling approach was used. Participants were recruited from three tertiary Grade‐A public general hospitals in Shanghai, China. The hospitals were selected purposively rather than randomly. Selection was based on three considerations:

1. all were tertiary Grade‐A public hospitals with comparable nursing management structures;
2. they represented different service areas within Shanghai; and
3. they were willing to provide administrative support for data collection.

Within each hospital, nursing administration offices assisted with internal coordination and circulation of the survey invitation across major departments and shifts. No probabilistic sampling procedure was used either at the hospital level or the participant level.

Eligibility criteria were as follows:

1. possession of a valid nurse licence;
2. at least 1 year of independent clinical experience; and
3. current full‐time employment at one of the participating hospitals

Nurses on leave or assigned to satellite campuses during the data collection period were excluded.

### 3.3 Sample Size Determination

The target sample size was determined before data collection based on practical SEM considerations. For covariance‐based SEM, a sample substantially above 200 is generally considered adequate for models of moderate complexity, and the present model included three latent constructs and a limited number of structural paths (Kline 2023). In addition, we aimed to secure a final analysable sample large enough to support stable CFA/SEM estimation and bootstrap mediation testing after excluding incomplete or invalid responses. On this basis, a recruitment target of approximately 450–500 completed responses was considered appropriate.

A total of 675 invitations were distributed, and 460 valid questionnaires were retained for analysis, yielding a response rate of 68.1%. This final sample size was judged adequate for the planned analyses.

### 3.4 Data Collection Procedure

Data were collected between September and November 2024 using a secure online survey platform commonly used in China. Nurses were invited through hospital intranet announcements and departmental communication channels.

The first survey page described the study purpose, voluntary nature of participation, confidentiality protections and data use. Participants provided electronic informed consent before proceeding.

To reduce careless responding, the questionnaire included response‐completeness checks and basic logic screening during data cleaning. Questionnaires with obvious invalid patterns were excluded. Because the online platform required responses to complete submission, there were no item‐level missing data among the retained valid questionnaires. Thus, no statistical imputation procedure was required for the final analytic dataset.

The study was approved by the institutional review board of REDACTED (Approval No. REDACTED, dated REDACTED) and adhered to the Declaration of Helsinki. No personally identifying information was collected and no material incentives were provided.

### 3.5 Measurement Instruments

All measures were self‐reported and based on previously validated instruments.

#### 3.5.1 Transformational Leadership

Transformational leadership was measured using the Multifactor Leadership Questionnaire (MLQ‐5X Short Form) developed by Bass and Avolio (1996). Items were rated on a 5‐point Likert scale from 1 (strongly disagree) to 5 (strongly agree). Higher scores indicated stronger perceived transformational leadership. Cronbach's alpha in this study was 0.87. A sample item is: ‘My supervisor articulates a compelling vision of the future’.

#### 3.5.2 Nurse Self‐Efficacy

Nurse self‐efficacy was measured using the Nursing Profession Self‐Efficacy Scale—Version 2 (NPSES‐2) (Magon et al. 2023). Items were rated on a 5‐point Likert scale from 1 (completely disagree) to 5 (completely agree). Higher scores indicated greater perceived nursing self‐efficacy. Cronbach's alpha in this study was 0.86. A sample item is: ‘I am confident in managing complex patient care situations independently’.

#### 3.5.3 Job Performance

Nurse job performance was measured using the Nurse Job Performance Scale developed by Wang et al. (2015), covering work coordination, work enthusiasm and work involvement. Items were rated on a 5‐point Likert scale, with higher scores indicating better self‐reported performance. Cronbach's alpha in this study was 0.86. A sample item is: ‘I coordinate well with colleagues to ensure smooth patient care delivery’.

All instruments underwent forward–backward translation and cultural adaptation in accordance with WHO guidance (World Health Organization 2016). A pilot test with 30 nurses supported clarity and comprehensibility.

### 3.6 Data Analysis

Data were analysed using SPSS 25.0 and AMOS 25.0.

First, descriptive statistics, skewness, kurtosis and Pearson correlations were calculated. Distributional diagnostics suggested no severe deviations from normality at the variable level (skewness < ±1.0; kurtosis < ±2.0). Multicollinearity was also examined through the correlation matrix and did not indicate problematic overlap among the main constructs.

Second, CFA was conducted to assess the measurement model. Latent constructs were modelled using item‐level indicators rather than parcelling, in order to preserve the dimensional integrity of the measures. Composite reliability (CR), average variance extracted (AVE) and discriminant validity were examined.

Third, covariance‐based SEM was used to test the hypothesised structural paths. In AMOS, models were estimated using the maximum likelihood (ML) estimator. Model fit was assessed using χ^2^/df, CFI, TLI, RMSEA and SRMR, following commonly used SEM reporting practices (Kline 2023).

Fourth, mediation was tested using bias‐corrected bootstrapping with 5000 resamples. An indirect effect was considered statistically significant when the 95% confidence interval did not include zero (Preacher and Hayes 2008).

Fifth, common method variance (CMV) was assessed using Harman's single‐factor test and an unmeasured latent methods factor model (Podsakoff et al. 2003). These procedures were used as diagnostic checks only and were not interpreted as eliminating the possibility of shared‐method bias.

Finally, several demographic variables (age, gender, education and tenure) were examined as potential controls in supplementary SEM analyses. Because these variables did not materially change the focal path estimates or improve overall model fit, they were not retained in the final parsimonious model. A summary of these supplementary analyses is reported in Appendix B.

In addition to the final partial mediation model, two alternative structural models were tested for comparison:

1. a full mediation model, in which the direct path from transformational leadership to job performance was constrained to zero; and
2. a direct‐effects model, in which transformational leadership and self‐efficacy were both specified as direct predictors of job performance, but the indirect pathway via self‐efficacy was not modelled as a mediation structure.

Comparisons were made using model fit indices and AIC values.

A significance level of *p* < 0.05 was used throughout.

## 4 Results

### 4.1 Participant Characteristics

Table 1 summarises the demographic characteristics of the 460 nurses included in the study. The sample was predominantly female (89.6%). Participants ranged in age from 20 to 55 years (M = 32.4, SD = 6.2). Most held a bachelor's degree (64.8%) and 64.1% had more than 5 years of work experience.

**TABLE 1:** Participant demographic characteristics.

Variable	Category	*n*	%
Sex	Male	48	10.4
	Female	412	89.6
Age	< 25	62	13.5
	25–34	238	51.7
	35–44	128	27.8
	≥ 45	32	7.0
Education	Junior college or below	124	27.0
	Bachelor's	298	64.8
	Master's or above	38	8.2
Work experience	1–5 years	165	35.9
	6–10 years	142	30.9
	> 10 years	153	33.2

These variables were examined in supplementary control‐variable models, but they were not retained in the final model because they did not substantially improve model fit or alter the substantive conclusions.

### 4.2 Descriptive Statistics and Correlation Matrix

Table 2 presents descriptive statistics and Pearson correlations among the three main variables. All correlations were positive and statistically significant. The strongest association was observed between nurse self‐efficacy and job performance (*r* = 0.52, *p* < 0.01).

**TABLE 2:** Descriptive statistics and correlation matrix.

Variable	M ± SD	1	2	3
1. Transformational leadership	3.82 ± 0.62	1		
2. Nurse self‐efficacy	4.05 ± 0.58	0.41^a^	1	
3. Nurse job performance	4.21 ± 0.43	0.38^a^	0.52^a^	1

^a^ *p* < 0.01 (two‐tailed).

### 4.3 Measurement Model Evaluation

CFA supported the adequacy of the measurement model. All factor loadings were statistically significant (*p* < 0.001) and exceeded 0.70, indicating satisfactory item reliability. The overall fit indices were acceptable:

χ^2^/df = 2.14.

CFI = 0.97.

TLI = 0.96.

RMSEA = 0.05.

SRMR = 0.04.

CR values exceeded 0.80, and AVE values exceeded 0.50 for all constructs, supporting convergent validity. Discriminant validity was also supported because the square roots of the AVEs exceeded the inter‐construct correlations.

Appendix A provides factor loading ranges, CR and AVE values.

Regarding CMV, the single‐factor model accounted for 29.4% of the variance, and the latent methods factor explained less than 20% of total variance. These results suggest that CMV was not overwhelmingly dominant, but they do not rule out possible inflation associated with same‐source self‐report measurement.

### 4.4 Structural Model and Hypothesis Testing

The final structural model explained 34% of the variance in nurse job performance (*R* ^2^ = 0.34). All three direct hypotheses were supported. As shown in Table 3, transformational leadership was positively associated with nurse self‐efficacy (Hypothesis 1: *β* = 0.33, *p* < 0.001), nurse self‐efficacy was positively associated with job performance (Hypothesis 2: *β* = 0.47, *p* < 0.001) and transformational leadership was positively associated with job performance (Hypothesis 3: *β* = 0.18, *p* < 0.001).

**TABLE 3:** Standardised path coefficients.

Path	*β*	SE	*t*	*p*	95% CI
Transformational leadership → self‐efficacy	0.33	0.05	6.60	< 0.001	[0.24, 0.42]
Nurse self‐efficacy → job performance	0.47	0.06	7.83	< 0.001	[0.35, 0.59]
Transformational leadership → job performance	0.18	0.04	4.50	< 0.001	[0.10, 0.26]

These findings indicate that both transformational leadership and self‐efficacy were statistically associated with self‐reported job performance, with self‐efficacy showing the stronger direct association.

### 4.5 Mediation Analysis

Bootstrapping with 5000 resamples showed that the indirect effect of transformational leadership on job performance through self‐efficacy was statistically significant, supporting Hypothesis 4. As presented in Table 4, the indirect effect was *β* = 0.15, with a 95% confidence interval of [0.09, 0.21]. This indirect path accounted for 46.3% of the total effect, indicating a pattern consistent with partial mediation.

**TABLE 4:** Mediation results.

Mediation path	Indirect effect	SE	95% CI	% Mediated
Leadership → self‐efficacy → performance	0.15	0.03	[0.09, 0.21]	46.3%

### 4.6 Alternative Model Comparison

Alternative structural models were tested to evaluate robustness. The partial mediation model (final model: χ^2^/df = 2.14, AIC = 10435.7) showed better fit than the full mediation model (χ^2^/df = 2.51, AIC = 10472.3) and the direct‐effects model (χ^2^/df = 3.12, AIC = 10588.6). The superiority of the partial mediation model (ΔAIC > 20) suggests that the observed data were more consistent with a model including both a direct association and an indirect association through self‐efficacy than with more restricted alternatives.

### 4.7 Structural Model Visualisation

**FIGURE 2:**
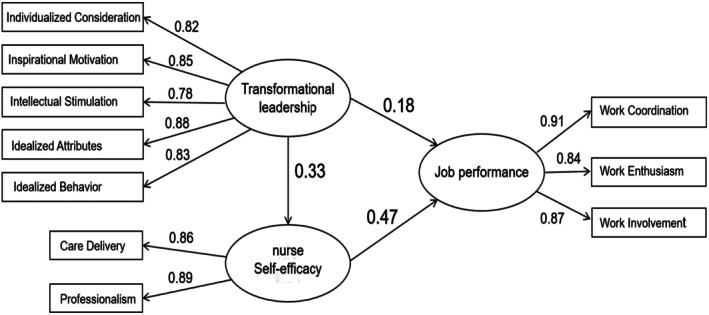
Structural equation model with standardised path coefficients. All item loadings ≥ 0.78; all paths significant at *p* < 0.001.

Figure 2 presents the final SEM with standardised coefficients. All observed indicators loaded strongly on their respective latent constructs, and all structural paths were statistically significant (*p* < 0.001). Self‐efficacy showed the strongest direct association with job performance in the final model.

## 5 Discussion

### 5.1 Summary of Main Findings

This study examined the associations among transformational leadership, nurse self‐efficacy and job performance among nurses in three tertiary Grade‐A public hospitals in Shanghai. Transformational leadership was positively associated with both nurse self‐efficacy and self‐reported job performance. Nurse self‐efficacy was also positively associated with job performance and showed a statistically significant partial mediating role in the relationship between transformational leadership and job performance.

Importantly, self‐efficacy emerged as the strongest direct predictor of job performance in the model. This finding is consistent with the view that nurses' confidence in their professional capability is meaningfully related to how they evaluate their work functioning in demanding clinical environments.

Because the study was cross‐sectional and based on same‐source self‐report, these findings should not be interpreted as demonstrating a causal mechanism. Rather, they identify an observed pattern of associations that is consistent with the proposed theoretical framework.

### 5.2 Comparison With Previous Studies

The positive association between transformational leadership and nurse self‐efficacy is consistent with prior work suggesting that supportive and motivating leadership behaviours are linked to stronger psychological resources among nursing staff (Niinihuhta et al. 2022; Wu et al. 2025). Similarly, the positive association between self‐efficacy and job performance aligns with previous nursing studies showing that nurses with greater confidence in their professional ability tend to report better work‐related functioning (Kim et al. 2022; Lee and Ko 2010).

The direct association between transformational leadership and job performance is also broadly consistent with earlier findings from nursing and healthcare research (Boamah et al. 2018; Hasan et al. 2023; Wang et al. 2021). Our results extend this literature by showing that, in this specific Shanghai tertiary hospital sample, self‐efficacy may represent one pathway through which these variables are statistically connected.

At the same time, our findings should be compared with prior studies cautiously because measures, settings and performance indicators vary across studies. In particular, many existing studies use different operationalisations of job performance or examine broader outcomes such as engagement or safety climate rather than self‐reported performance alone.

### 5.3 Cultural and Organizational Context

The Chinese tertiary hospital context is relevant to interpreting these findings. Such hospitals often operate within relatively formal hierarchies and high‐demand working conditions, which may increase the salience of leadership cues for frontline nurses (Hu et al. 2025; Ying et al. 2021). In these settings, transformational leadership behaviours may be interpreted not only as interpersonal support but also as signals of organisational endorsement and professional recognition.

This contextual interpretation is theoretically plausible, but it was not directly tested in the present study. Variables such as power distance orientation, collectivist values, perceived organisational support and unit climate were not measured. Therefore, any cultural interpretation should remain tentative and should be examined more explicitly in future research.

### 5.4 Theoretical Implications

This study offers several theoretical contributions.

First, it applies Bandura's social cognitive theory and the JD‐R model in a unified framework within a Chinese hospital sample. The results are consistent with the idea that leadership can function as a job resource and that self‐efficacy can function as a personal resource associated with job performance.

Second, the observed partial mediation pattern suggests that transformational leadership may be linked with job performance both directly and indirectly through self‐efficacy. This supports a more nuanced understanding of leadership processes than a simple direct‐effect model.

Third, the study contributes context‐specific evidence from three Shanghai tertiary public hospitals. Although the findings should not be generalised to all nurses in China, they provide useful evidence from an important and understudied organisational setting.

### 5.5 Practical Implications

Several practical implications follow from these findings, while bearing in mind their associational nature.

First, nursing administrators may consider strengthening transformational leadership competencies such as vision communication, individualised support, constructive feedback, and role modelling. These leadership behaviours were positively associated with nurse self‐efficacy and performance in this sample.

Second, nursing education and management programmes may benefit from incorporating self‐efficacy‐enhancing strategies such as simulation training, structured mentoring, feedback and guided reflection. Because self‐efficacy showed the strongest direct association with performance, these strategies may be useful targets for professional development.

Third, hospital managers may consider reinforcing supportive work structures, including regular feedback mechanisms, recognition systems and opportunities for professional growth. Although this study did not test interventions, the findings are consistent with the view that supportive leadership and psychological resources matter in demanding hospital settings.

These implications should be interpreted prudently. The present study does not demonstrate that implementing such strategies will necessarily cause performance improvements, but it does identify practical areas that are reasonably aligned with the observed associations and with prior theory.

### 5.6 Limitations and Directions for Future Research

This study has several limitations.

First, the cross‐sectional design does not allow causal inference or temporal ordering. Although the mediation pattern was theoretically informed and statistically supported, it should be interpreted as associational only. Longitudinal, multi‐wave, or intervention‐based designs are needed to examine temporal dynamics more rigorously.

Second, all focal variables were measured using single‐source self‐report. This is particularly important for the outcome variable, job performance, which was assessed by nurse self‐report rather than supervisor ratings, peer ratings or objective indicators. Although CMV diagnostics did not suggest overwhelming method bias, these tests do not fully resolve the concern that same‐source measurement may inflate observed associations. Future studies should use multi‐source designs and, where feasible, combine survey and administrative data.

Third, sampling was based on convenience recruitment from only three tertiary Grade‐A public hospitals in Shanghai. Therefore, the findings should not be considered representative of all nurses in China or of all hospital types. External validity may be limited for rural hospitals, private hospitals, specialty hospitals or settings with different organisational cultures.

Fourth, the participating hospitals shared broadly similar institutional characteristics, which may have reduced contextual heterogeneity. Future studies should compare multiple regions and hospital types and should consider multilevel models that incorporate unit‐level or hospital‐level influences.

Fifth, several contextual variables were not included, such as department type, shift pattern, staffing levels, organisational climate and cultural value orientations. These may shape the strength of the associations observed here and should be incorporated into future moderated or multilevel mediation research.

## 6 Conclusion

This study examined the associations among transformational leadership, nurse self‐efficacy, and self‐reported job performance among nurses in three tertiary Grade‐A public hospitals in Shanghai, China. Transformational leadership was positively associated with self‐efficacy and job performance and self‐efficacy showed a statistically significant partial mediating role.

These findings are consistent with an integrated social cognitive theory and JD‐R perspective in which leadership and personal resources are jointly relevant to work outcomes. However, because the data were cross‐sectional, convenience‐based and derived from single‐source self‐report, the findings should be interpreted as evidence of association rather than causation.

Overall, this study provides context‐specific evidence from Shanghai tertiary public hospitals and suggests that transformational leadership and nurse self‐efficacy are worthwhile variables for continued investigation in nursing management research. Future studies using longitudinal, multi‐source and more diverse sampling designs are needed to clarify temporal ordering, improve external validity and test contextual moderators more directly.

## Funding

The author has nothing to report.

## Ethics Statement

This study was approved by the Joint Ethics Committee of REDACTED (approval number: REDACTED). This study adhered to the Declaration of Helsinki.

## Consent

Written informed consent was obtained from all participants prior to data collection.

## Conflicts of Interest

The author declares no conflicts of interest.

## Data Availability

The data that support the findings of this study are available on request from the corresponding author. The data are not publicly available due to privacy or ethical restrictions.

## Appendix A See Table A1

**TABLE A1:** Confirmatory factor analysis results for the measurement model.

Construct	Item code(s)	Factor loadings	Composite reliability (CR)	Average variance extracted (AVE)
Transformational leadership	TL1–TL5	0.78–0.89	0.89	0.63
Nurse Self‐efficacy	SE1–SE6	0.79–0.90	0.88	0.61
Job performance	JP1–JP5	0.80–0.88	0.87	0.60

*Note:* All standardised factor loadings are significant at *p* < 0.001.

## Appendix B Summary of Supplementary Analyses

### Control‐Variable Models

Age, gender, education and work experience were entered as controls in supplementary SEM analyses. Their inclusion did not materially change the magnitude or significance of the focal structural paths, nor did they improve model fit meaningfully. They were therefore excluded from the final model for parsimony.

### Alternative Models

Full mediation model: direct path from transformational leadership to job performance constrained to zero.

Direct‐effects model: direct paths retained, but the mediation structure not specified as the final indirect model.

The partial mediation model showed the best overall fit.

### CMV Diagnostics

Harman's single‐factor test and the latent methods factor model did not indicate that a single factor dominated the covariance structure, but these tests were treated as diagnostic rather than definitive.
